# Patient experiences of recovery after heart valve replacement: suffering weakness, struggling to resume normality

**DOI:** 10.1186/1472-6955-12-23

**Published:** 2013-09-26

**Authors:** Selina Kikkenborg Berg, Ann-Dorthe Zwisler, Birthe D Pedersen, Katrine Haase, Kirstine Lærum Sibilitz

**Affiliations:** 1 Rigshospitalet, The Heart Center, University of Copenhagen, Copenhagen, Denmark; 2 Gentofte Hospital, Cardiology, University of Copenhagen, Copenhagen, Denmark; 3 Research Unit of Nursing, Institute of Clinical Research, Faculty of Health Sciences, University of Southern Denmark, Odense, Denmark

**Keywords:** Heart valve disease, Transcatheter aortic valve implantation, Valve surgery, Patient experience, Recovery

## Abstract

**Background:**

Heart valve disease is becoming a public health problem due to increasing life expectancy and new treatment methods. Patients are at risk of developing depression, anxiety or post-traumatic stress disorder after heart valve surgery. To better plan proper care, describing and understanding patients’ perception of recovery after heart valve replacement is essential. The objective was to describe the experience of recovery at home after heart valve replacement.

**Methods:**

Qualitative interviews were conducted with 10 patients representing the population and these were later transcribed. The analysis was inspired by Ricoeur’s theory of interpretation, which consists of three levels: naive reading, structured analysis, and critical interpretation and discussion.

**Results:**

The overall concept that emerged was *suffering weakness and struggling to resume normality*. Patients all struggled to resume normal living, both in regaining physical strength and in reestablishing balance in overall living. The overall concept can be interpreted in terms of the following themes: *Disturbed network*: *Invaluable relatives, Contact with healthcare staff, Rehabilitation*. *Disturbed body*: *Stressful complications, Bodily attention, Physically affected, Physical capability*. *Recovery*: *Interrupted living, Suffering weakness, Gradual recovery, Achieving normality*. *Reflections*: *Thoughts about the procedure* and *Feeling sad and fragile*.

**Conclusion:**

The study presents the main themes of network, body, recovery and reflection for ten patients after heart valve replacement. These main themes can overall be summarized as suffering weakness and struggling to resume normality. Patients felt weak with a changed body, but after a long recovery process regained vitality and returned to their daily life.

## Background

Heart valve disease is becoming a public health problem due to increasing life expectancy and new treatment methods [1,2]. Heart surgery can be a challenging and stressful life-event [3], however, patient experiences after heart valve surgery are inadequately described.

With heart valve disease progression, symptoms develop, including dyspnoea, fatigue, syncope and palpitations. Once symptomatic, heart valve disease severely restricts the performance of daily living and heart valve surgery often is inevitable [4]. Until recently, surgical valve replacement with open heart surgery was the only effective treatment option. Though, this procedure is associated with higher morbidity and mortality in the elderly compared to younger patients. Thus, surgical valve replacement is not always suitable for elder patients [5]. Interventional cardiology, such as Transcatheter Aortic Valve Implantation (TAVI) techniques, has emerged as an alternative treatment to open-heart surgery to elder patients [6]. Procedural success, faster recovery and favourable clinical outcomes in the short term have been reported [7]. Overall mortality does not differ significantly between TAVI and traditional heart surgery, and duration of hospital stay is equal [8]. However, length of stay in intensive care unit is shorter for TAVI patients, though, 30 day mortality and risk of major stroke seems to be increased for the TAVI group [9].

Regardless the surgical technique, post-surgery, co-morbidity affects quality of life [10,11], and fragile patients are at risk of developing depression, anxiety or post-traumatic stress disorder [12,13]. Patients can feel changed during their illness, leading to lifelong fragility [14]. Also, at discharge and returning home the feeling of vulnerability and worries about transition phases appear, and a fear of information gap can be dominant [15]. To better plan proper after care, describing and understanding patients’ perception of recovery after heart valve replacement is essential.

Knowledge in the field of patient experiences after heart valve surgery is still lacking, especially studies focusing solely on patients after heart valve surgery. Therefore, the present study aimed to explore patient experiences of health recovering heart valve replacement, both traditional open-heart surgery and transcatheter valve replacement. This was done by having them describe experiences of daily living after hospital discharge, including current health problems and thoughts about the future, which will be useful when developing clinical guidelines concerning after care for this growing group of patients.

### Scientific framework

The study is set within a phenomenological-hermeneutic frame, as it deals with human perception and experiences of recovering from heart valve replacement. The study is based on the French philosopher Paul Ricoeur’s (1913–2005) approach to hermeneutic- phenomenology, his theory of interpretation, and his theory of time and narrative [16,17]. The approach places this present study in line with a number of scientific works inspired by the above-mentioned theories [18,19]. In Ricoeur’s writings, he tries, through reflection and argumentation, to merge different understandings and sciences, and through this dialectic process gain new understanding and insight, and to find a third way [16-18]. Phenomenology is a theory of what appears in one’s consciousness. Husserl is considered to be the founder of phenomenology and described the term "intentionality", and what it means to go "straight to the point" by giving a pure description of a phenomenon as it appears to the conscious mind. This calls for open ended questions to allow for a direct and free way of expression. Human experiences are non-directly expressed through language, and require interpretation, which is why Ricoeur expanded Husserl’s term phenomenology by involving hermeneutics, which concerns understanding and interpretation of the written word [16]. Ricoeur’s understanding entails both explanation and understanding, and is set within a dialectic process from what is said to what is spoken about. This happens in one process, and according to Ricoeur, the correlation between explanation and understanding, between understanding and explanation, is ultimately "the hermeneutic circle" [16]. Ricoeur’s theory concerns how we as individuals become aware of our participation in the world by expressing it. To retell an event means to bring the past into the present in order to shape the future. By expressing meaning, as it manifests itself in the recovery process, it is possible to become aware of patients’ needs in order to plan for proper after care.

### Purpose

The purpose of the study is to describe patients’ experience of recovery at home after heart valve replacement. The qualitative investigation aimed to answer the following research questions: What are the experiences of returning home after heart valve replacement? What are the new experiences in daily living? Which health problems are experienced? What are the thoughts about the future? The questions were also used as an interview guide, in order to fulfil the purpose of the study.

## Methods

A qualitative research method with a phenomenological-hermeneutic approach was used. A semi structured interview guide was developed to allow for open-ended questions, asking the patients to describe their experiences freely and openly according to the scientific frame.

### Setting and subjects

In this study, ten patients who had a heart valve replacement within the previous six to nine months were included. The time frame of six to nine months was chosen to ensure time to experience recovery. Two patients had pulmonary valve replacement, and eight had aortic valve replacement, either with traditional surgery or TAVI (Table 1). We decided that ten patients would provide a good insight into how the recovery could be experienced [20,21], knowing that including more patients could potentially add to the level of insight. Interviewees were selected from the hospital database to represent both genders, different age groups, and type of replacement (traditional surgery or TAVI) to achieve maximum variation [22]. Gender differences have been found in rehabilitation outcomes [23,24], which is why both genders should be represented. Furthermore, age could have an impact, as well as type of replacement, as open heart surgery has more physical impact than TAVI. The ten patients were included consecutively. A research assistant initially approached the participants by giving them letters, followed by phone calls to ask patients if they would participate. An informed consent form was signed before beginning the interview. The demographic details of the interviewees are presented in Table 1.

**Table 1:** Demographic data of interviewees (n = 10)

	**n**
**Male gender**	5
**Age (mean/range)**	66 (20–85)
**Live with partner**	8
**Employed**	3
**Valve operated on**	
**aorta/pulmonary**	8/2
**Open surgery/TAVI**	5/5
**Rehabilitation**	5

TAVI: Transcatheter Aortic Valve Implantation. Rehabilitation: patients were asked if they were offered and participated in rehabilitation at their local setting.

### Data collection

The pre-understanding of the authors as nurses was that recovery would be smooth but long and that patients were not followed closely by the health care system. At the interviews these pre-understandings were set aside as no close-endend questions were asked and patients were allowed to talk about the topics they found to be important. In this way we seeked to open the interviewees world up to us. All participating patients underwent an interview in an office at the hospital or in their homes. Few of them invited us to their homes, to avoid transportation to the hospital. A semi-structured interview guide was developed to ensure consistency and encourage openness and flexibility during the interview. While it provided a frame, it was not followed step by step. The patients spent a few moments reading through the guide before the interview to familiarize themselves with the questions. The interviews took 30–50 minutes on average. Two researchers were present during the interviews, one being the primary interviewer. Patients were informed about this in the initial letter. We have previously used this practice [25], and learned that by using two interviewers, it is possible to utilise their abilities to bring different nuances to light. We did not find it too overwhelming for patients and experienced depth and openness in their responses. When reading the transcripts we saw how the questions from the second interviewer added to the exploration. The researchers were not known to the patients beforehand. The interviews were tape recorded and subsequently transcribed by a secretary. Data is kept confidential and is reported anonymous.

### Analysis

In accordance with the method inspired by Ricoeur [18,19], the analysis consisted of three levels. These levels were naive reading, structured analysis, and critical interpretation and discussion. The two interviewers analyzed separately and thereafter discussed the findings on each level.

#### Naive reading

The text was read several times in order to grasp its meaning as a whole. The interpreters read the text with a phenomenological attitude that requires them to be open enough to allow the text to speak to them, putting the pre-understanding aside. The naive reading is regarded as a first conjecture and it has to be validated or invalidated by the subsequent structural analysis [19,26]. The first thoughts about what is said is noted.

#### Structural analysis

In order to validate or invalidate the naive reading we moved from what the text says to what it speaks about. First by identify all units of meaning, that is parts in the text were the patient describe the experience of recovery at home after heart valve replacement and next by identifying patterns and formulating units of significance and themes [18,19]. First, we read the whole text and divided it into units of meaning (what is said about the experience of recovery at home after heart valve replacement). Second, the units of meaning were reflected upon in relation to the naive understanding. The validation process consists of making sure that the interpretation of what the text speaks about is true to the original spoken words. Then the units of meaning were sorted and condensed, and units of significance were formulated (what is talked about). Contradictory data formed units of significance that were not value loaded in order to include different patient perspectives on the same matter. This means that we did not label a unit e.g. "lack of rehabilitation" but only "rehabilitation" when contradictory data was found e.g. some experienced lack of rehabilitation some were happy about the rehabilitation program. Next, units of significance were condensed even more and themes were formulated. A theme is a thread of meaning that penetrates text parts. A theme identifies an essential meaning of a lived experience; these themes are formulated as condensed descriptions and abstract concepts [19]. The themes were reflected on against the background of the naive understanding to see whether the themes validated or invalidated the naive understanding. When the structural analysis invalidated the naive understanding, the whole text was read again and a new naive understanding was formulated and checked by a new structural analysis. This process of comparing the naive reading and the structural analysis was repeated until the naive understanding was validated through the dialectic process between explanation and understanding [18,19]*.*

#### Critical interpretation and discussion

The themes were compared and contrasted to other research findings and the meaning discussed.

### Ethical considerations

Patients were asked to participate in the study verbally and they confirmed their willingness in writing. They had time to reflect on participation in the study before written informed consent was given. All data material was treated in confidence and patients were assured anonymity. The study followed the recommendations of the Declaration of Helsinki II [27].

## Results and Discussion

The overall concept that emerged from the analysis was *suffering weakness, struggling to resume normality*. Patients had felt weak since even before they needed the operation. Weakness was very present in a more physical form after the procedure, where some experienced extreme tiredness and deconditioning. Patients all struggled to resume normal living, both in regaining physical strength, and in reestablishing balance in overall living. This means that they struggle to reestablish their role in society and in their network and strive to regain emotional and physical balance.

The overall concept *suffering weakness, struggling to resume normality* can be interpreted in terms of the following themes: Disturbed network (Table 2), Disturbed body (Table 3), Recovery (Table 4) and Reflections (Table 5). These themes consist of different significant units comprising different units of meaning. The overall findings are presented in a table below each theme. The tables show the results as well as examples of meaning unit "quotes" to demonstrate the analysis process. Meaning units were chosen using the criteria of being representative, short and precisely formulated. Interpretation and discussion follow below the tables.

**Table 2:** Disturbed network

**Meaning units**	**Significant units**	**Theme**
*"If it wasn’t for my husband it would not have gone so well", "Without my wife I could not have returned home so soon", "I bothered my friends with it, so I got a kind of processing, psychologically".*	Invaluable relatives	Disturbed network
*"I needed someone to talk to about how to do things", "They had my back", "They didn’t want me to come".*	Contact with healthcare staff	
*"Nice to start up in a safe and good environment", "It did not fit my working schedule", "Sad it wasn’t offered".*	Rehabilitation	

**Table 3:** Disturbed body

**Meaning units**	**Significant units**	**Theme**
*"It turned out to be a blood clot in my leg, I am not who I once was", "they said it was stroke", "I am still afraid that something breaks loose".*	Stressful complications	Disturbed body
*"I am very aware of my body now", "I am afraid of doing a lot of things, thinking how my heart reacts", "I compensate for my scar by working out, to build muscles".*	Bodily attention	
*I don’t lose my breath…it is better and better", "I lose my breath as much as before","I improved my exercise capacity by 37 % but I didn’t feel it".*	Physical capability	
*"I lost 10 kg", "Food tasted awkward", "I slept 5 times a day", "I still had a lot of pain in the body".*	Physically affected	

**Table 4:** Recovery

**Meaning units**	**Significant units**	**Theme**
*"It was very intrusive in my life", "It was not as it used to be, I was not the girlfriend I used to be", "I had to just follow my body and sleep".*	Interrupted living	Recovery
*"When I came home, I couldn’t do anything", "I felt weak when I walked on the street, I felt everyone pushed me", "I was limited, had to just follow the body".*	Suffering weakness	
*"I felt better every day", "It was a big step when I took a walk alone, a little further every day", "I was really happy to experience improvements every week".*	Gradual recovery	
*"I feel as I normally do", "It was a statement to them" "When I can go to the gym it’s not that bad".*	Achieving normality	

**Table 5:** Reflections

**Meaning units**	**Significant units**	**Theme**
*"I did not regret having it, but I hope it lasts many years", "I expect to have the valve replaced again, but that is ok", "I think my life has been prolonged 2–3 years".*	Thoughts about the procedure	Reflections
*"There is death anxiety", "I lost my buoyancy, it was too hard, I didn’t care if I lived or died", "You get depressed sometimes, you are a bit fragile inside".*	Feeling sad and fragile	

### Disturbed network

The meaning units and significant units leading to the theme Disturbed network are presented in Table 2. Being in a fragile situation after heart valve surgery, network became of great importance, as has also been shown for ischemic heart disease [28]. Often patients could not take care of themselves after hospital release and spoke about *invaluable relatives*. The relationships changed for a while; parents, partners and friends were given a new role as helpers both physically and emotionally, changes that have also been described in other cardiac operated patients [3]. Specifically, preparing food, assisting in heavy lifting, driving etc. were tasks that patients were assisted with. Some expressed that they could not have returned home so soon if it wasn’t for the help of their spouse. Also, in processing all their experiences patients needed friends and family to listen and share views. Many felt grateful towards the relatives. However some relatives did not handle the changed situation so well, often due to broken expectations and some relationships had to be repaired and built up again after recovery. This pattern is also described in the literature as the process of having a changed relationship to the partner for a while can be a tremendous challenge for relatives and spouses [29].

Patients needed guidance and answers in trying to interpret symptoms and sensations after hospital release, which is a familiar experience after heart surgery [30]. Some experienced good *contact with healthcare staff* while others felt rejected and "lost". Insecurity about how to handle illness and recovery were often present, especially at the beginning, when the safe hospital environment was replaced with home, a feeling that also applies to spouses [31].

Some felt confused about whether follow-up treatment took place at the treating hospital or the local hospital. Some patients were offered *rehabilitation* while others were not, or the offer came too late and did not fit their working hours. Resuming a normal life was important. As reported in observational and randomized clinical studies, participation in a heart disease rehabilitation program contributes to a sense of safety, and patients benefit regarding physical ability and gain knowledge by sharing their experience with healthcare staff and co-patients [32-34]. Those not offered rehabilitation were regretful. One turned it down and tried to make her own way ahead. Needing physical rehabilitation to recover was an opportunity to get physically active for patients not previously active. Some were still active after the program ended, and others returned to a more inactive way of living.

### Disturbed body

The meaning units and significant units leading to the theme Disturbed body are presented in Table 3. Many patients experienced *stressful complications,* e.g. thrombosis in the brain, kidney or leg, pericardial exudates, pseudoaneurism, or anaemia. It affected them physically, but they also lived in fear of further complications, such as stroke. Some experienced fear and anxiety of how to be active in a safe way. Keeping the heart safe was a major concern. They became very aware of the body and paid attention to signals and sensations. Fragility has been shown to be the essence of the experiences of open heart surgery. Anxiety and vulnerability comprising possible complications and a body which is no longer to be trusted are prior findings for this patient group leading to fragility [14]. The *bodily attention* also focused on the scar, which for some was psychologically stressful. Some compensated for the scarred body by training more to develop a more fit body. Thoughts were made as to how others would look at the scar and some wished to cover it up, not only to protect it from the sun, but also to make a partner feel more comfortable during intimacy. Disturbance to body image due to sternotomy has previously been described. A decline in feelings of sexual desirability and intimacy was associated with the scar and caused the feelings of distance from spouse or partner [35]. One study showed that women generally associated their scar with feelings of mutilation and damage. Acceptance of the scar came with time and as it faded. Participants suggested that their life experience and older age contributed to their ability to accept and adapt to the scarring [36]. *Physical capability* differed after the procedure. Some felt they had benefitted tremendously by not being short of breath and having more energy in physical activities, others were very disappointed in not feeling improvements, even though health care professionals sometimes could measure an improvement. Many were *physically affected*, experienced a great weight loss, and were very affected by the procedure. A newly published study suggests that a higher pre-operative fat-free mass index in patients undergoing cardiac surgery indicates better ability to cope with post-operative stress, resulting in fewer complications. In addition, post-operative loss of muscle mass was associated with decreased vitality [37]. Strange metallic tastes in the mouth were confusing and connected to continuous food loathing. Severe sleeping problems within the first 1–2 months were reported. Disturbed sleep associated with hospitalization and recovery after treatment of cardiac conditions has been well described [38-40]. Pain was also a major issue for those with open heart surgery, a familiar phenomenon in several studies [41,42].

### Recovery

The meaning units and significant units leading to the theme Recovery are presented in Table 4. Patients felt that the operation was very inconvenient. *Interrupted living* meant that plans and hopes for the future had to be changed, e.g. travelling and family planning. The state of going through disturbance in life, and how disease and hospitalization interrupt living, has previously been described [25]. The weakness and illness patients felt also disturbed normal living. *Suffering weakness* meant feeling incapable, not capable of doing normal activities and resuming the daily life. An enormous tiredness overwhelmed many patients. Being a weak person was also experienced when walking the streets and being afraid of people bumping into the chest, or taking the bus and being afraid of sudden braking. Patients underestimated how long usual activities required e.g., how long a bicycle ride would take or how much work they could do, thus not recognising their own poor physical state. However, quality of life studies generally have observed an acceptable level of self-perceived health after heart valve surgery, the challenge, therefore is to identify patients with difficulties and recovering incompletely, either physically or mentally [43,44].

Most patients experienced *gradual recovery.* Every day or week meant new joyful progress. Just walking the street alone was experienced as a tremendous achievement. Recovery was fast for some while others were affected for many months. Patients described how they got colour in the cheeks again after a few weeks and struggled their way back to *achieve normality*. This process also includes returning to work, which can be a challenge after heart valve surgery and take several weeks [45].

Feeling normal, not ill, was the goal, and in that process to regain independence and a normal relationship to relatives. For some, demonstrating normality was important, not to be labelled a disabled person. When normal activities were resumed, patients felt relieved and proud of their recovery.

### Reflections

The meaning units and significant units leading to the theme Reflections are presented in Table 5. Many *thoughts about the procedure* were experienced. Feeling relieved in everyday activities, being able to do more without losing breath was important results of the operation. Some felt disappointed that they did not feel any difference. Despite experiencing life threatening complications, patients did not regret having the procedure. Some expected to have to go through it again at some point, but that was okay for them. However patients who underwent open heart surgery hoped for a more gentle method should future intervention be necessary. Some felt their life was prolonged. Thinking about the seriousness of the event could lead to patients *feeling sad and fragile*. Fragility seems an aggressive, continuous and recurring theme and feeling for this patient group [14], [46]. During recovery thoughts about what really happened in hospital or what could have happened occurred. Thinking about the closeness of death and the disturbing experiences the relatives had was heart breaking. In struggling for both psychical and emotional recovery some were very depressed and almost gave up at times. Feeling fragile after heart surgery is well described, and predisposed individuals have a higher risk of suffering depression, anxiety and even post-traumatic stress disorder even months after heart valve surgery [12,46-48]. For others the experience was not too stressful and they quickly achieved emotional closure.

### Clinical implications

When planning discharge, follow-up care and rehabilitation programs for patients who have gone through heart valve replacement, the themes identified on meaning of network, bodily implications, recovery and reflections on meaning of life should be integrated as important focus areas to ensure that patients can return to normal life after the intervention.

Up to now guidelines concerning follow-up care after heart valve replacement have mainly focused on physical rehabilitation [49], however, this study indicates that rehabilitation for this group of patients should also include a psycho-educative element to cover full physical as well as mental recovery. Supportive contact with healthcare professionals seems beneficial [50], and in rural areas, telemedicine could be the answer. Specific topics need attention when it comes to preparing for the recovery; sleep, pain and fatigue issues, bodily changes and needs, the psychological impact of the procedure and overall feeling of fragility, the pattern and time frame of recovery, and preparing the relatives for the temporary changes in the family. Also, the study shows the importance of the connection between specialized treatment and aftercare. Entering a rehabilitation program would probably be beneficial for this patient group within a few weeks after hospital discharge.

### Trustworthiness

Within the hermeneutical qualitative methodology, the following criteria can be used to assess the study’s trustworthiness: credibility, transferability, dependability and conformability [51].

Credibility is a parallel to internal validity, securing a match between the realities of the interviewees and the study results. The patients’ statements were repeated to them during the interview to ensure that the interpretation was correct. Furthermore, we used two interviewers to increase the ability to explore the patients’ experiences and we used two researchers to conduct the analysis. Two interviewers could possibly be overwhelming to the patients and it is possible that "one to one" interview would create more intimacy and openness. However we did not experience it to be a problem that two interviewers were present. When reading the transcripts it became clear that the second interviewer were able to bring out new perspectives and more detail that the first interviewer missed in the process of both listening and asking elaborating questions. Furthermore most interviews took place at the hospital, a location that for some patients represents a lifesaving setting to which they feel gratitude. That could have influenced the results too. Other interviews took place at the patients’ home which might have provided a sense of security to speak more critically about the recovery process and health care system. To ensure an appropriate assessment of transferability, relevant information e.g. time, place, demographic data and cardiac characteristics of the interviewees were presented, as were the inclusion criteria. The dependability criterion deals with the traceable and documentable research process established and secondly on the conformability of data. In this paper relevant information about background, methodology, methods, processes and analyses is presented. Conformability, within this type of research, is related to the integrity of the findings that are rooted in the data. In this paper the process of analysis is described and relevant parts are presented. Quotes, meaning units, leading back to the interviewees support each finding.

The results do not clarify differences between operative procedures, age and gender that could all potentially lead to differences in recovery. Further studies are needed to explore this.

## Conclusions

The purpose of the study was to describe patients’ experience of recovery at home after heart valve replacement. Four main themes was identified namely Disturbed network, Disturbed body, Recovery and Reflections. These main themes can overall be summarized as *suffering weakness, struggling to resume normality*. Patients felt weak with a changed body, but after a long process during which they felt fragile, gained vitality and returned to their daily life.

With the continuous advancement of heart valve surgery, increasing number of patients undergoing heart valve surgery, rising age at time of surgery and recognition of patients needs - both physical and mental - after heart valve surgery, it is ever more important to plan proper after care to prevent morbidity and readmissions to hospital.

## Competing interests

The authors declare that they have no competing interests.

## Authors’ contributions

SKB in collaboration with ADZ, BDP, KH and KLS designed the study. SKB and KH conducted the interviews and analyses. SKB drafted the manuscript. All revised the manuscript critically. All have given their final approval of the version to be published.

## Pre-publication history

The pre-publication history for this paper can be accessed here:

http://www.biomedcentral.com/1472-6955/12/23/prepub
